# Reversible impairment of the *ku80* gene by a recyclable marker in *Aspergillus aculeatus*

**DOI:** 10.1186/2191-0855-3-4

**Published:** 2013-01-12

**Authors:** Shuji Tani, Atsushi Tsuji, Emi Kunitake, Jun-ichi Sumitani, Takashi Kawaguchi

**Affiliations:** 1 Graduate School of Life and Environmental Sciences, Osaka Prefecture University, 1-1 Gakuen-cho, Naka-ku, Sakai, Osaka, 599-8531, Japan; 2 Present address: Industrial Research Institute of Ishikawa, 2-1 Kuratsuki, Kanazawa, Ishikawa, 920-8203, Japan

**Keywords:** *Aspergillus aculeatus*, Homologous recombination, *Ku* gene, Counter selection, Marker recycling

## Abstract

Auxotrophic mutants of *Aspergillus* can be isolated in the presence of counter-selective compounds, but the process is laborious. We developed a method to enable reversible impairment of the *ku80* gene (*Aaku80*) in the imperfect fungus *Aspergillus aculeatus*. *Aaku80* was replaced with a selection marker, orotidine 5’-phosphate decarboxylase (*pyrG*), followed by excision of *pyrG* between direct repeats (DR) to yield the *Aaku80* deletion mutant (MR12). The gene-targeting efficiency at the ornithine carbamoyltransferase (*argB*) locus was drastically elevated from 3% to 96% in MR12. The frequency of marker recycling depended on DR length. One uridine auxotroph was obtained from 3.3 × 10^5^, 1.4 × 10^5^, and 9.2 × 10^3^ conidia from strains harboring 20-, 98-, and 495-bp DRs, respectively. Because these strains maintained the short DRs after 5 d of cultivation, we investigated whether *Aaku80* function was disrupted by *pyrG* insertion with the 20-bp DR and restored after excision of *pyrG*. The *Aaku80* disruption mutant (coku80) was bred by inserting *pyrG* sandwiched between 20-bp DRs into the second intron of *Aaku80*, followed by excision of *pyrG* between the DRs to yield the coku80rec strain. Analyses of homologous recombination frequency and methyl methanesulfonate sensitivity demonstrated that *Aaku80* function was disrupted in coku80 but restored in coku80rec. Furthermore, *pyrG* was maintained in coku80 at least for ten generations. These data indicated that reversible impairment of *ku80* in *A*. *aculeatus* is useful for functional genomics in cases where genetic segregation is not feasible.

## Introduction

*Aspergillus aculeatus* [NBRC 108796], a filamentous fungus isolated from the soil, produces a number of extracellular cellulose- and hemicellulose-degrading enzymes that exhibit synergism with *Trichoderma* cellulases. Alkali-treated rice straw was shown to be hydrolyzed almost completely into monosaccharides by mixed-culture filtrate of these two species (Murao et al. [1979,1988]). Previously, we constructed a host–vector system of *A*. *aculeatus* that expressed the *A*. *aculeatus* β-mannosidase gene (Kanamasa et al. [2003]). The *A*. *aculeatus* expression system yielded a 10-fold higher level of the enzyme than did an *Aspergillus oryzae* expression system, indicating the high potential of *A*. *aculeatus* as a factory to produce useful proteins (Kanamasa et al. [2007]). However, the concerted action of several cellulases, each with different properties, is considered to be essential for the effective hydrolysis of cellulose into monosaccharides. To breed *A*. *aculeatus* that simultaneously produces many types of cellulases, a marker-recycling technique, enabling to excise a selection marker gene from transformants without leaving any exogenous DNA fragments, must be developed (Akada et al. [2006]). This technique enables the excision of a selection marker gene from transformants without leaving exogenous DNA fragments; multiple cellulase genes can be introduced into a host strain with a limited number of selection markers. Furthermore, elucidating how the expressions of the cellulose-degrading enzyme-encoding genes are regulated will improve enzyme production. Therefore, one must develop highly-efficient homologous recombination (HR) techniques as well as a marker-recycling technique in the imperfect fungus *A*. *aculeatus*.

Recently, deletion of the *ku70*/*80* or *lig4* genes, which are both involved in DNA double strand repair, was demonstrated to elevate the frequency of HR in filamentous fungi (Ishibashi et al. [2006]; Krappmann et al. [2006]; Mizutani et al. [2008]; Ninomiya et al. [2004]; Takahashi et al. [2006]). These achievements enabled the study of the cellular roles of interesting genes. Based on this information, we cloned and replaced the *A*. *aculeatus ku80* gene with a selection marker to yield the *A*. *aculeatus ku80* deletion mutant. We also investigated the length of the direct repeats (DRs) required for efficient marker recycling in the *A*. *aculeatus ku80* deletion mutant to enable a reversible impairment of *ku80*.

## Materials and methods

### Strains

All *A*. *aculeatus* strains used in this study were derived from wild-type *A*. *aculeatus* no. F-50. Unless otherwise stated, all strains were propagated at 30°C in appropriately supplemented minimal medium (MM) (Adachi et al. [2009]). The *pyrG* (orotidine 5’-phosphate decarboxylase gene)-deficient mutants (uridine auxotrophs) were spontaneously isolated by spreading 1 × 10^6^*A*. *aculeatus* wild-type conidia on a plate containing 1 mM 5-fluoroorotic acid (5-FOA) and 0.4 mM uridine. Colonies that showed a 5-FOA-resistant phenotype were isolated and transformed with the *Aspergillus nidulans pyrG* gene (*AnpyrG*), and strains that showed uridine prototrophy were regarded as *pyrG*-deficient mutants (pyrG13, uridine auxotrophs), which were used as hosts to breed arginine prototrophs and the *ku80* deletion mutant in *A*. *aculeatus*.

### Nucleic acid analysis

PCR was performed using PrimeSTAR HS DNA polymerase (TaKaRa, Kyoto, Japan) as described in the manufacturer’s instructions, except that the annealing temperature was 55°C and there were 30 PCR cycles. All primers used in this study are shown in Table 1. Genomic DNA was isolated from ground mycelia with Micro Smash MS-100R (Tomy, Tokyo, Japan) as described previously (Adachi et al. [2009]). Southern hybridization was performed as described previously (Adachi et al. [2009]).

**Table 1:** List of primers used in this study

	
Primers used to clone the *Aaku80* segment	
ku80F1	5’-ATGGCNGANAAGGAAGCAACNGT-3’
ku80R3	5’-CGTCCATAYTCATANCCYTTGGC-3’
Primers used to clone the *AaargB* segment	
AaargBF3	5’-CARGCNATHGCNGAYTTYCARAC-3’
AaargBR2	5’-GGNARRCARTGCATRAAYYTCCA-3’
Primers used for the *Aaku80* deletion	
aku80AF1	5’-GCGGCCGCATGGTTCTATGTCGCTTGGC-3’
aku80AR2	5’-CGACTGGGTCTCTGCGATGGCGAGTAGCGACGAAG-3’
aku80BF1	5’-TGTTTATTGCAGCCAGCATGATGGTGCCAACGCAG-3’
aku80BR1	5’-GCGGCCGCATTGCGATGCTGGGTGAC-3’
aku80CF1	5’-CTTCGTCGCTACTCGCCATCGCAGAGACCCAGTCG-3’
aku80CR1	5’-GAGAGCATTGTCTGCGGATGGTGGGGATGGGTTG-3’
npyrF2	5’-CAACCCATCCCCACCATCCGCAGACAATGCTCTC-3’
npyrR1	5’-CTGCGTTGGCACCATCATGCTGGCTGCAATAAACA-3’
Primers used for the *A*. *nidulans pyrG* insertion into *Aaku80*	
coku80NF	5’-TAGATATCAGCTCTTCTTGCCATCGC-3’
coku80NR	5’-TTAGCGGCCGCTGAAACCATCAGTCTTGTCTGC-3’
coku80CF	5’-GCCTTAATTAAGAGTCCCACGAAGCGATG-3’
coku80CR	5’-TGCTCTAGATGTCGTCATCTTGATAGCGA-3’
copyrF	5’-TAGCGGCCGCAATGCTCTCTATC-3’
copyrR	5’-CCTTAATTAACCGTTACACATTTCCACTCA-3’
Primers used for the *AaargB* disruption	
AaargB5F	5’-GGCGTGATTCTTTGCTCC-3’
AaargB5r2	5’-GAGAGCATTGTCTGCGGGGTCGGCGTCAGGTC-3’
AaargB3f3	5’-GAGTGGAAATGTGTAACGGATTGCATGGACCCGAG-3’
AaargB3r	5’-GGGGACATGGCTTCCTAC-3’
npyrF3	5’-GACCTGACGCCGACCCCGCAGACAATGCTCTC-3’
npyrR3	5’-CTCGGGTCCATGCAATATGCTGGCTGCAATAAAC-3’
mrarg5F	5’-GGCGGGCTGGAGCTCAC-3’
mrarg5iR	5’-GCACCGCGCTAGCCGGTCATTCG-3’
mrarg5iF	5’-CGAATGACCGGATAGCGCGGTGC-3’
mrarg5R	5’-GAGAGCATTGTCTGCGGGCGATCTTGAGGCCTTCCAG-3’
mrpyr5F	5’-CTGGAAGGCCTCAAGATCGCCCGCAGACAATGCTCTC-3’
mrpyr5R	5’-GACAAAATCCTCGCTCTCCTC-3’
mrarg3F1	5’-CGGTATTGACTAAAAGGGATCTGGAAGGCCTCAAGATCGC-3’
mrarg3F2	5’-CGGTATTGACTAAAAGGGATCCGCTGCAGGCCATC-3’
mrarg3F3	5’-CGGTATTGACTAAAAGGGATGCGCCATCTGCTCTCG-3’
mrarg3R	5’-GAAGGTCAAGGCCGGTG-3’
mrpyr3F	5’-TGCCCCTCCAGGATAAC-3’
mrpyr3R1	5’-GCGATCTTGAGGCCTTCCAGATCCCTTTTAGTCAATACCG-3’
mrpyr3R2	5’-GATGGCCTGCAGCGGATCCCTTTAGTCAATACCG-3’
mrpyr3R3	5’-CGAGAGCAGATGGCGCATCCCTTTTAGTCAATACCG-3’
Primers used to amplify DNA probes for Southern blot analyses	
ku80R4	5’-GGAACCCAGAAGAATACGACAC-3’
AaargB3f3	5’-GAGTGGAAATGTGTAACGGATTGCATGGACCCGAG-3’
AaargB3r	5’-GGGGACATGGCTTCCTAC-3’
DxlnR-F1	5’-GTCGACCACCGCGATCCAGCAGTACGCCA-3’
DxlnR-R2	5’-CACAATCCACAGTGAGACCCACTCTCTCGC-3’

### Cloning the *A*. *aculeatus ku80* and ornithine carbamoyltransferase genes

A part of the *A*. *aculeatus ku80* gene (*Aaku80*) was amplified from the *A*. *aculeatus* genome by PCR using the primers ku80F1 and ku80R3, which were designed to anneal to the region conserved among the *Aspergillus ku80* genes. The 1.2-kb PCR product was inserted into the *Eco*RV site of pBluescript II KS (+) (pBS II KS (+)) (Stratagene, La Jolla, CA, USA), and both strands were sequenced twice. Southern blot analysis of the *A*. *aculeatus* genome using the *Aaku80* segment as a DNA probe suggested that a 6-kb genomic DNA fragment digested with *Sph*I possessed the *Aaku80* gene. This fragment was then cloned into the *Sph*I site of pUC118 (Takara) to yield pAaku80.

The *A*. *aculeatus* ornithine carbamoyltransferase gene (*AaargB*) was also cloned from the *A*. *aculeatus* genome by the same strategy. The *AaargB* segment was amplified with the primers AaargBF3 and AaargBR2. After performing Southern blot analysis and colony hybridization using the *AaargB* segment as a DNA probe, the *AaargB* open reading frame (ORF) with flanking regions, an approximately 6-kb *Hin*dIII-digested fragment, was cloned into pBS II KS (+), yielding pAaargB.

### Transformation of *A*. *aculeatus*

The transformations were performed by following the protoplast–PEG method as described previously (Adachi et al. [2009]). The strains were propagated at 30°C in MM supplemented with the appropriate nutrients.

### Gene disruption and marker recycling

**Figure 1:**
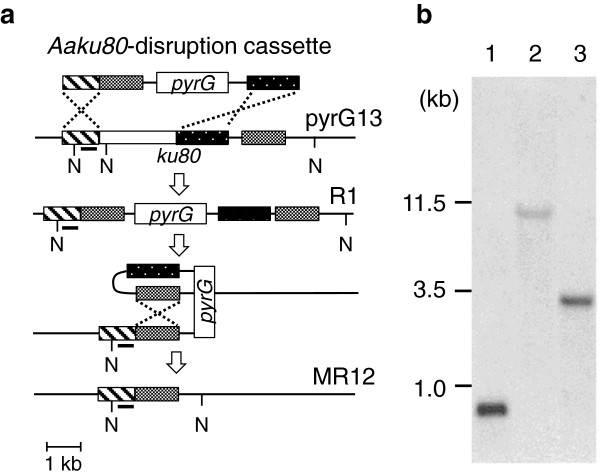
**Southern blot analysis of the *Aaku80*-deletion mutants.** (**a**) Restriction enzyme maps of *Aaku80* loci in the pyrG13, R1, and MR12 strains of *Aspergillus aculeatus*. The DNA probe used for Southern blot analysis is indicated with solid bars. N, restriction sites of *Nco*I. (**b**) Results of the Southern blot analysis using genomic DNA digested with *Nco*I (N). Lanes 1, 2, and 3 are pyrG13, R1, and MR12, respectively.

The linear DNA fragments used for the gene deletion of *Aaku80* followed by marker recycling were generated by fusion PCR as described previously (Akada et al. [2006]). To construct the *Aaku80* deletion cassette, the 5’ and 3’ regions of the *Aaku80* gene, which are required for HR, were amplified from *A*. *aculeatus* genomic DNA using the primer pairs aku80AF1 and aku80AR2 for the 5’ region and aku80BF1 and aku80BR1 for the 3’ region. The 3’ flanking region of the *Aaku80* gene, which formed DRs, was amplified from *A*. *aculeatus* genomic DNA using the primer pair aku80CF1 and aku80CR1. The *AnpyrG* fragment was amplified using pPL6 (Punt et al. [1991]) as a template with the primer pair npyrF2 and npyrR1. The four fragments were joined by fusion PCR using the primers aku80AF1 and aku80BR1, and the fused product was subcloned into the *Eco*RV site of pBS II KS (+), which yielded pDku80. The *Aaku80* deletion cassette was amplified with the primers aku80AF1 and Aku80BR1 using pDku80 as a template (Figure 1). The fragments were introduced into the pyrG13 strain using the protoplast–PEG method. The deletion of endogenous *Aaku80* was confirmed by Southern blot analysis using DNA probes that were amplified with the primers ku80R4 and aku80BR1 using pAaku80 as a template. FOA-resistant strains by marker recycling were spontaneously isolated from 1 × 10^4^ to 10^6^*A*. *aculeatus* transformant conidia in MM with 1 mM FOA and 0.4 mM uridine. These conidia were purified by repeating mono-spore isolation twice on the plates to obtain the conidia of homokaryons. Their growth was examined on plates with or without 8 mM uridine.

To develop a strain enabling a reversible impairment of *Aaku80*, an *Aaku80* disruption cassette 2 was constructed. The 5’- and 3’-regions of the *Aaku80* gene were amplified from *A*. *aculeatus* genomic DNA using the primer pairs coku80NF and coku80NR for the 5’ region and coku80CF and coku80CR for the 3’ region. The second intronic region was amplified with the coku80NR and coku80CF primers. The *AnpyrG* fragment was amplified using pPL6 as a template with the primer pair copyrF and copyrR. The 5’-side of *Aaku80* digested with *Eco*RV and *Not*I and *AnpyrG* digested with *Not*I were inserted into the *Eco*RV site in pBS II KS (+) to yield pCO5P. The 3’-side of *Aaku80* digested with *Pac*I, and *Xba*I was ligated to the *Pac*I and *Xba*I sites on pCO5P to yield pCO5P3. The *Aaku80* disruption cassette 2 was amplified with the primers coku80NF and coku80CR using pCO5P3 as a template. The fragments were introduced into the pyrG13 strain using the protoplast–PEG method. The disruption of endogenous *Aaku80* was confirmed by Southern blot analysis using DNA probes that were amplified with the primers ku80R4 and aku80BR1 using pAaku80 as a template.

Homologous recombination frequencies were determined by simply replacing the *AaargB* gene with *AnpyrG* in pyrG13 and the *A*. *aculeatus ku80* deletion-mutant (MR12). To construct the *AaargB* deletion cassette 1, the 5’ (981 bp) and 3’ (1,034 bp) regions of the *AaargB* gene were amplified from *A*. *aculeatus* genomic DNA using the primer pairs AaargB5F and AaargB5r2 for the 5’ region and AaargB3f3 and AaargB3r for the 3’ region. The *AnpyrG* fragment was amplified using pPL6 as a template with the primer pair npyrF3 and npyrR3. The three fragments were joined by fusion PCR using the primers AaargB5f and AaargB3r, and the fused product was subcloned into the *Eco*RV site of pBS II KS (+) to yield pDAaargB. The *AaargB* deletion cassette 1 was amplified with the primers AaargB5f and AaargB3r using pDAaargB as a template. The deletion of endogenous *AaargB* was confirmed by Southern blot analysis using DNA probes that were amplified with the primers AaargB3f3 and AaargB3r using pAaargB as a template.

To retain the *AaargB* sequence in the arginine auxotroph, the *AaargB* function was disrupted by insertion mutagenesis. This mutation was introduced by overlapping two fragments amplified by primer sets mrarg5F and mrarg5iR and mrarg5if and mrarg5R. These fragments were fused to the 5’-side of the *AnpyrG* fragment (−456 to 683 nt from the translation start site of *AnpyrG*) amplified with primer set mrpyr5F and mrpyr5R to yield the 5’arg-pyr fragment. The 3’-side of *AaargB* fragments with different DR lengths were amplified with the primer sets mrarg3F1 and mrarg3R, mrarg3F2 and mrarg3R, and mrarg3F3 and mrarg3R, respectively. These fragments were fused to the 3’-side of the *AnpyrG* fragment (174 to 925 nt from the translation start site) amplified with the primer sets mrpyr3F and mrpyr3R1, mrpyr3F and mrpyr3R2, and mrpyr3F and mrpyr3R3 to yield the argB3B1, argB3B2, and argB3B3 fragments, respectively. In the last step, the 5’arg-pyr fragment was fused to the argB3B1, argB3B2, and argB3B3 fragments by amplifying with the primer set mrarg5F and mrarg3R, which yielded the argBDR-20, argBDR-98, and argBDR-495 fragments, respectively. These fragments were introduced into the pyrG13 strain using the protoplast–PEG method. The disruption of endogenous *AaargB* was confirmed by Southern blot analysis using DNA probes that were amplified with the primers mrarg5F and mrarg5iR for the 5’-DNA probe and mrarg3F1 and mrarg3R for the 3’-DNA probe using pAaargB as a template.

Deletion of the *A*. *aculeatus xlnR* gene, encoding a gene-specific activator controlling cellulase and hemicellulase gene expression, was performed as described previously (Tani et al. [2012]). The *xlnR* deletion in the selected strains was confirmed by analyzing each genome using Southern blot analysis and PCR using the primers DxlnR-F1 and DxlnR-R2.

## Results

### Deletion of the *Aaku80* gene and recycling of the *AnpyrG* marker

As a first step toward developing HR and marker-recycling systems in *A*. *aculeatus*, *Aaku80* was cloned. Sequence analysis and a homology search revealed that the *Aaku80* gene comprised a 2,711-bp ORF interrupted by nine introns and encoding 726 amino acid residues with a calculated molecular mass of 81,088 Da (DDBJ Acc. no. AB741874). The *Aaku80* gene in pyrG13 (*Aaku80*^+^, *pyrG*^–^) was replaced with *AnpyrG* using the *Aaku80* deletion cassette (Figure 1a). Forty transformants were obtained by selection on a uridine-free medium. Southern blot analysis confirmed that one (2.5%) of these transformants, designated R1, had one copy of the deletion cassette inserted into the *Aaku80* locus (Figure 1b, lane 2). Next, we tried to excise the *AnpyrG*-containing fragment sandwiched by a pair of 1-kb DRs to reuse *AnpyrG* as a selection marker, as is commonly done in *Saccharomyces cerevisiae* (Akada, et al. [2006]). Conidia (approximately 1 × 10^4^) were spread onto a 5-FOA plate, and 20–30 colonies on average that should have been 5-FOA-resistant appeared after 5 d of cultivation, while it took more than 10 d for resistant mutants to emerge on 5-FOA plates by spontaneous mutation. After mono-spore isolation, more than 90% of the isolates showed uridine auxotrophy. Southern blot analysis showed that the expected excision event of intramolecular HR occurred in 9 of 10 uridine auxotrophs (Figure 1b, lane 3). One strain (*Aaku80*^–^, *pyrG*^–^) was designated as strain MR12.

### Characteristics of the *Aaku80* disruptant

**Figure 2:**
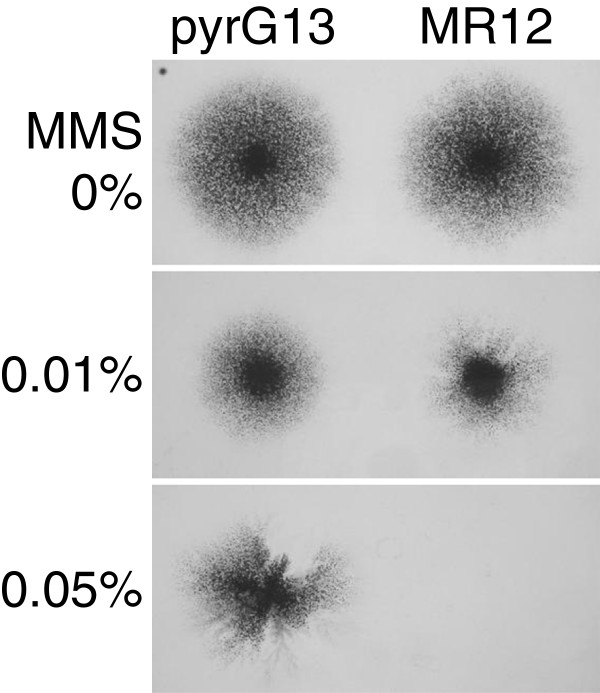
**Sensitivity of pyrG13 (*pyrG***^**–**^**) and MR12 (*Aaku80***^**–**^**, *pyrG***^**–**^**) strains of *Aspergillus aculeatus *to methyl methanesulfonate.** Conidia were spotted and grew on minimal medium with or without methyl methanesulfonate (MMS) for 5 d.

The growth and sporulation of MR12 on MM were indistinguishable from those of the control strain (pyrG13), but its growth on 0.05% methyl methanesulfonate (MMS) was poorer than that of pyrG13 (Figure 2). This result agreed well with previous reports that *ku* disruption in *Neurospora crassa* or *Aspergillus fumigatus* increased sensitivity to the DNA-damaging reagent (Ninomiya et al. [2004]; da Silva Ferreira et al. [2006]).

To compare the HR frequency at the *AaargB* locus in pyrG13 with that in MR12, the *AaargB* gene was cloned. Sequence analysis and a homology search revealed that *AaargB* comprised a 1,089-bp ORF with no introns and encoded 362 amino acid residues with a calculated molecular mass of 38,980 Da (DDBJ Acc. no. AB741875). The *AaargB* gene was replaced with a selection marker using the *AaargB* deletion cassette 1 in pyrG13 and MR12 to elucidate the gene targeting frequencies. Arginine auxotrophs were found in one of 32 transformants of pyrG13 (targeting frequency of 3%) and in 29 of 31 transformants of MR12 (94%). Southern blot analysis showed that all arginine auxotrophs derived from MR12 had a single copy of the cassette at the target locus by double HR (data not shown).

### Construction of *pyrG argB* double-auxotrophic host and effect of DR length on intramolecular homologous recombination

**Figure 3:**
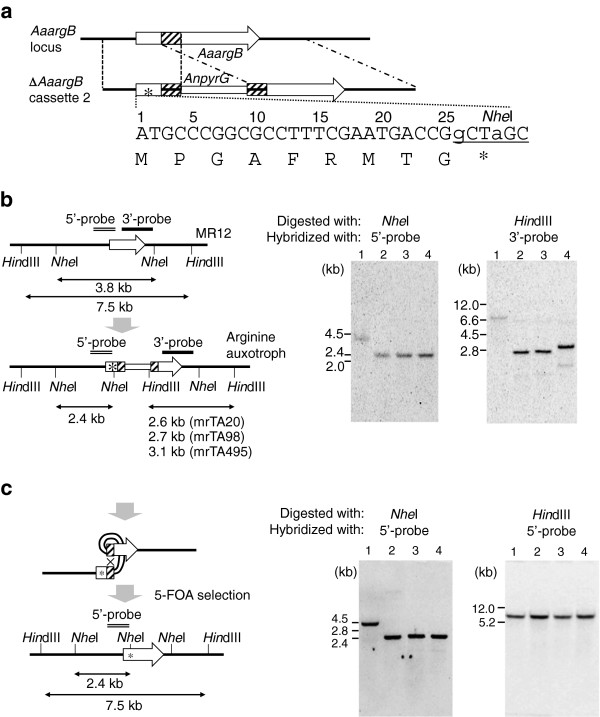
**Southern blot analyses of the *argB*-auxotroph mutants harboring different lengths of direct repeats for marker recycling.** (**a**) Diagram of the *AaargB* disruption cassette 2. Thecandy-striped bar indicates the direct repeat sequences. The insertion of guanine (g) and adenine (a) yielded, respectively, a *Nhe*I restriction site and a stop codon (asterisk). (**b**) Left: restriction enzyme maps of the *AaargB* loci in the MR12 strain and its transformants with the *AaargB* disruption cassette 2. The DNA probes used for Southern blot analysis are indicated with a double line (5’-probe) and a solid bar (3’-probe). Right: results of Southern blot analysis using genomic DNA digested with *Nhe*I and *Hin*dIII. Lanes 1, 2, 3, and 4 are MR12, mrTA20, mrTA98, and mrTA495, respectively. (**c**) Left: restriction enzyme maps of the *AaargB* locus after the *pyrG* excision by intramolecular homologous recombination. Right: Southern blot analyses were performed as in (b). Lanes 1, 2, 3, and 4 are MR12 and FOA-resistant strains from mrTA20, mrTA98, and mrTA495, respectively.

The arginine auxotrophic host was generated with point mutations to retain the *argB* ORF as the target of homologous transformation. The *argB* disruption cassette 2 was designed to insert the nucleotides G and A behind the 25th G and 27th T from a translation start site, which yielded a *Nhe*I recognition site and Ala9Gly mutation followed by a stop codon, respectively (Figure 3a). The disruption cassettes were also designed to harbor different length DRs (20, 98, or 495 bp) and the *AnpyrG* selection marker to elucidate the effect of DR length on intramolecule HR. The cassettes were introduced into MR12 and yielded 15, 10, and 20 uridine prototrophic transformants, respectively. From these, 11 (73%), 10 (100%), and 17 (85%) arginine auxotrophs were isolated, respectively. Strains having a single copy of the insert were selected to give the mrTA20, mrTA98, and mrTA495 strains harboring the DRs of the indicated lengths (Figure 3b, lanes 2–4). Conidia (1 × 10^6^) of mrTA495 were spread on plates containing 5-FOA, uridine, and arginine, and 120 colonies developed. Ten 5-FOA-resistant strains were selected for further analysis and found to be uridine auxotrophs. Southern blot analysis confirmed that *AnpyrG* was excised by intramolecular HR from the *argB* locus in nine of the ten uridine auxotrophs (Figure 3c, lanes 2–4). Thus, an *A*. *aculeatus* host with double selection markers was successfully constructed. The frequency of the emergence of the uridine auxotroph by intramolecular HR from mrTA495 was calculated to be 1.1 × 10^–4^. Those in mrTA98 and mrTA20 were calculated to be 7.0 × 10^–6^ and 3.0 × 10^–6^, respectively (Table 2). These results indicated that excision frequency was correlated with longer DRs. Although the frequency of *AnpyrG* excision in mrTA20 was almost equal to the frequency of isolating the uridine auxotroph by spontaneous mutation from the wild-type strain, these events could be distinguished by culture duration, because the 5-FOA resistant strains resulting from intramolecular HR appeared earlier than did those by spontaneous mutations.

**Table 2:** Frequencies of intramolecular homologous recombination between direct repeats (DRs) of different lengths

**Strain**	**mrTA20**	**mrTA98**	**mrTA495**
Length of DR (bp)	20	98	495
No. of 5-FOA-resistant strains	6	14	120
No. of resistant strains analyzed	6	6	10
No. of uridine auxotrophs	6	4	9
No. of homologous recombination events between DRs	3	3	9

The 5-fluoroorotic acid (5-FOA) resistant strains were isolated from 1.0 × 10^6^ conidia.

### Mitotic stability analysis of DNA with direct repeat sequences

Subculturing the mrTA495 strain five times on MM with uridine did not increase the frequency of emergence of 5-FOA resistants (data not shown). Furthermore, intramolecular recombination between DRs was investigated by Southern blot analysis using chromosomal DNA isolated from the mrTA495 strain grown in liquid MM with or without uridine for 5 d. The hybridization patterns of the *AaargB* locus in the mrTA495 were the same as shown in Figure 3b (lane 4). The same results were obtained in mrTA20 (lane 2) and mrTA98 (lane 3). These data indicated that the short DRs were stably maintained in the transformants.

### Insertion of *AnpyrG* into the second intron of *Aaku80* enables the disruption and restoration of *Aaku80*

**Figure 4:**
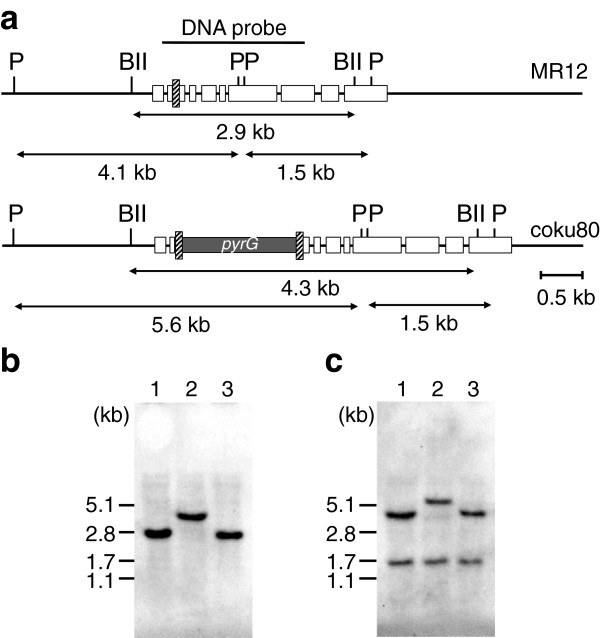
**Southern blot analyses of the *Aaku80*-disruption mutants.** (**a**) Diagram of *Aaku80* disruption cassette 2. The candy-striped bars indicate the second intron of *Aaku80*. Results of Southern blot analyses using genomic DNA digested with (**b**) *Bgl*II (BII) and (**c**) *Pst*I (P). Lanes 1, 2, and 3 are MR12, coku80, and coku80rec strains, respectively.

Establishing a simple method to restore the function of *Aaku80* after deleting specific genomic regions would be an advantage in filamentous fungi without a feasible genetic segregation technique, such as *A*. *aculeatus*. Therefore, we designed the *Aaku80* disruption cassette 2, which introduced the *AnpyrG* gene into the second intron of the *Aaku80* gene (Figure 4a). Because the direction of *AnpyrG* transcription is the same as that of *Aaku80*, we presumed that the termination of *AnpyrG* transcription yielded immature transcripts of *Aaku80* and resulted in the loss of Ku80 function. Furthermore, we expected that a functional Ku80 protein would be produced even if a frameshift mutation occurred between the repeats because the insert was in an intron in *Aaku80*. The pyrG13 strain was transformed with the *Aaku80* disruption cassette 2 and yielded transformants harboring the cassette integrated as a single copy, namely coku80, into the *Aaku80* locus by homologous integration (Figure 4b, lane 2). The coku80rec strain was isolated by excision of the *AnpyrG* gene by marker recycling at the frequency of 3.3 × 10^–5^ (Figure 4b, lane 3). Sequence analyses of *Aaku80* of five independently-isolated coku80rec strains confirmed that *AnpyrG* was excised between DRs without any mutations.

Growth and sporulation of the coku80 and coku80rec strains on MM were indistinguishable from those of MR12 (*Aaku80*^–^, *pyrG*^–^) and pyrG13 (*pyrG*^–^), but growth of coku80 on 0.05% MMS was as poor as that of MR12. However, both coku80rec and pyrG13 grew normally on 0.05% MMS (data not shown). Transformation of pyrG13, MR12, coku80, and coku80rec with the *xlnR* deletion cassette yielded 1 (of 51 transformants; 2%), 31 (of 46; 67%), 26 (of 35; 74%), and 1 (of 38; 3%) xylan-utilization-deficient mutants. *AnpyrG* insertion into the second intron of *Aaku80* successfully disrupted *Aaku80* function, which was restored by intramolecular HR between 20-bp DRs. Furthermore, the coku80 strain stably maintained the *AnpyrG* gene after 10 generations and yielded 17 xylan-utilization-deficient mutants out of 21 transformants (81%). These data indicate that coku80 is a feasible host for genetic analysis of interesting genes in the *Aaku80* functional background.

## Discussion

In *Aspergillus*, auxotrophic mutants, such as *pyrG*, *niaD* (nitrate reductase), or *sC* (ATP-sulfurylase)-deficient mutants, can be simply isolated because they are positively selected in the presence of the counter-selective compounds ([Arst 1968]; Daboussi et al. [1989]; Goosen et al. [1987]). To advance molecular biology in *A*. *aculeatus*, we designed and validated a HR method for gene replacement by deleting the *Aaku80* gene. The *Aaku80* deletion drastically increased the gene-targeting frequency from 3% to 94% and enabled the construction of double auxotrophic mutants (*Aaku80*, *argB*^–^, *pyrG*^–^). This drastic increase in gene-targeting frequency reduced the labor required to isolate auxotrophic mutants, such as an arginine auxotroph, without the use of counter-selective compounds. The 94% targeting frequency in *A*. *aculeatus* was high enough for practical use in gene replacement. However, the targeting frequency for approximately 1-kb homologous sequences was lower than in *N*. *crassa* (Ninomiya et al. [2004]). Because homology length is critical to the homologous integration frequency, *A*. *aculeatus* might require a longer homologous region for gene targeting.

Because the number of marker genes is limited, establishing a marker-recycling technique is necessary for the consecutive insertion of gene expression cassettes or multiple-gene deletions. For marker rescue, the *creloxP* recombination system of bacteriophage P1 has been shown to mediate efficient recombination between 34-bp *loxP* sites flanking a marker gene in yeast and filamentous fungi, resulting in excision of the marker gene (Krappmann et al. [2005]; [Sauer 1987]). Gene deletion by HR between the *loxP* sites results in marker removal, leaving behind a single repeat at the deleted gene locus. Therefore, developing a self-cloning technique to breed genetically-modified filamentous fungi is important. To achieve this aim, the double-marker enrichment technique was adopted for target-gene deletion in *A*. *fumigatus*, *Aspergillus awamori*, *A*. *nidulans*, and *Leptosphaeria maculans* (d’[Enfert 1996]; [Gardiner and Howlett 2004]; [Krappmann and Braus 2003]; Michielse et al. [2005]). In this strategy, a second selectable marker is added to the gene deletion cassette to distinguish between HR and non-homologous recombination. Upon HR, the second selection marker is lost, whereas after integration by non-homologous recombination, transformants carrying both selectable markers are obtained. Alternatively, once a mutant with a two-way (positive and negative) selectable marker such as *pyrG* is isolated, marker recycling enables not only consecutive gene deletion, but also consecutive gene insertion without leaving behind an exogenous DNA fragment. Although 50–100 bp of DRs were sufficient to facilitate gene-replacement cassette integration at the homologous locus at a gene replacement efficiency of 50–100% in *Schizosaccharomyces pombe* and *Saccharomyces cerevisiae* (Bahler et al. [1998]; Wach et al. [1994]), longer DRs were thought to be required in filamentous fungi ([Hynes 1996]; [Maruyama and Kitamoto 2008]; Nielsen et al. [2008]; Ninomiya et al. [2004]). However, our results indicated that the correct excision event could occur even with a 20-bp DR, although the frequency was relatively low. The observation that a shorter DR works sufficiently for correct excision should help to design a deletion cassette for marker recycling. Using this method, we are constructing an *A*. *aculeatus* host with multiple selectable markers to enable the breeding of filamentous fungi that produce several different cellulases to hydrolyze cellulosic substances with high efficiency.

Despite whole-genome sequencing, the roles of many gene products in fungi remain unknown, in part because gene deletion may not alter the phenotype when more than one paralog exists in the genome. The technique developed in this paper could be a useful tool for post-genomic research.

## Competing interests

The authors declare that they have no competing interests.
